# Analysis of the application of a gene chip method for detecting *Mycobacterium tuberculosis* drug resistance in clinical specimens: a retrospective study

**DOI:** 10.1038/s41598-021-97559-y

**Published:** 2021-09-09

**Authors:** Gang Feng, Wenhao Han, Jinyan Shi, Rongrong Xia, Jianchun Xu

**Affiliations:** 1 The Fourth People’s Hospital of Lianyungang City, Lianyungang, 222000 Jiangsu Province China; 2 Haibin Rehabilitation Hospital, Lianyungang, Jiangsu Province China

**Keywords:** Biological techniques, Microbiology

## Abstract

Most *Mycobacterium tuberculosis* (*Mtb*) resistant to rifampicin (RIF) has mutations in the *rpoB* gene, while most *Mtb* resistant to isoniazid (INH) has mutations in the *katG* gene or *inhA* promoter. We used gene chip technology to detect mutations in these genes to determine the resistance of *Mtb* to RIF and INH. A total of 4148 clinical specimens with sputum smear positivity for acid-fast bacilli (AFB) were detected. Then, taking the results of the drug sensitivity test (DST) as the reference standard, the detection efficiency of sputum samples from different grades of positive smears was compared in detail. We found that the sensitivity of the gene chip method for detecting sputum samples with a grade ≥ AFB 2 + was higher than that of sputum samples with a grade ≤ AFB 1 + (P < 0.05). When the grade of the sample was ≤ AFB 1 +, the sensitivity of the gene chip method was 72.6% for RIF, 67.3% for INH, and 60.0% for MDR-TB. When the grade of the sample was ≥ AFB 2 +, the sensitivity of the gene chip method was 84.5% for RIF, 78.2% for INH, and 73.9% for MDR-TB. The results show that gene chip technology can be directly used to diagnose drug-resistant tuberculosis in clinical specimens, and the diagnostic efficiency for the detection of sputum specimens with a grade ≥ AFB 2 + is better than that of other sputum specimens.

## Introduction

Tuberculosis (TB) is a serious global public health problem caused by *Mtb*. It is an infectious disease that greatly endangers human health. Since rifampicin was first introduced as an anti-TB drug in 1972, standardized treatment regimens have been used to treat TB for nearly half a century; however, *Mtb* still threatens the health of nearly one-third of the world's population, many of whom will suffer from the disease during their lives^1^. Globally, there were 10 million new TB cases in 2019, and 1.4 million people died of TB^2^. China is one of the countries that is the most threatened by TB^3^. Governments need to invest a large amount of money and manpower to prevent and treat TB every year. Although new and highly effective bacillus Calmette-Guérin (BCG) vaccines have been used in newborns and young children as vaccinations to prevent TB, they cannot effectively prevent adults from being infected with *Mtb*^4^. Drug treatment is still very important for the prevention and treatment of TB. More seriously, the occurrence and prevalence of drug-resistant TB, especially multidrug-resistant TB (MDR-TB), is a serious threat to TB prevention and treatment in China. According to the World Health Organization Tuberculosis Report (2020), in 2019 alone, approximately 546,000 patients in China were infected with MDR-TB. And China is one of the three countries with the largest burden worldwide.

Drug-resistant TB can be classified as primary resistance or secondary resistance. Primary resistance is caused by the direct infection of patients with drug-resistant *Mtb*, while secondary resistance is caused by the acquisition of drug resistance abilities after the infection of people with drug-resistant strains due to factors such as drug treatment^5^. MDR-TB is a disease caused by *Mtb* that is resistant to at least the two of the most commonly used first-line anti-TB drugs, RIF, and INH^6^. Many mechanisms cause drug resistance in *Mtb*, but most of the drug resistance in clinical *Mtb* strains is due to chromosomal mutations^7^. Studies have shown that there are many types of gene mutations in drug-resistant TB. Among them, *rpoB* gene mutations account for 95–99% of RIF-resistant strains; among the strains resistant to INH, *katG* gene mutations account for 60–95%, and *inhA* promoter mutations account for 8–43%^8^.

Generally, the culture-based conventional drug sensitivity test has long been considered the gold standard for diagnosing drug-resistant *Mtb,* although it is time-consuming and labour-intensive^9^. Traditional *Mtb* drug susceptibility tests can take 4–8 weeks or even longer to obtain TB drug susceptibility test results, which obviously does not allow for the early treatment of drug-resistant TB^10,11^. The diagnosis time is too long, leading to inappropriate medication, which not only increases the treatment time and the patient's financial burden but also may lead to more serious drug resistance, resulting in the aggravation of the disease and even death among patients^12–14^. With the advancement of science and technology, a new solution to this problem has been developed. In recent years, a variety of molecular biology techniques have been applied for the detection of drug resistance in *Mtb*^15–17^. Examples include loop-mediated isothermal amplification (LAMP), simultaneous amplification testing (SAT), Xpert MTB/RIF (Cepheid, Sunnyvale, CA), MTBDRplus, TB-Biochip and TB-Biochip-2 technologies (Moscow, Russia)^18^. CapitalBio (Beijing, China) developed a DNA microarray chip method based on a variety of molecular analyses with PCR and reverse hybridization to detect drug resistance in TB bacteria^19^.

**Figure 1:**
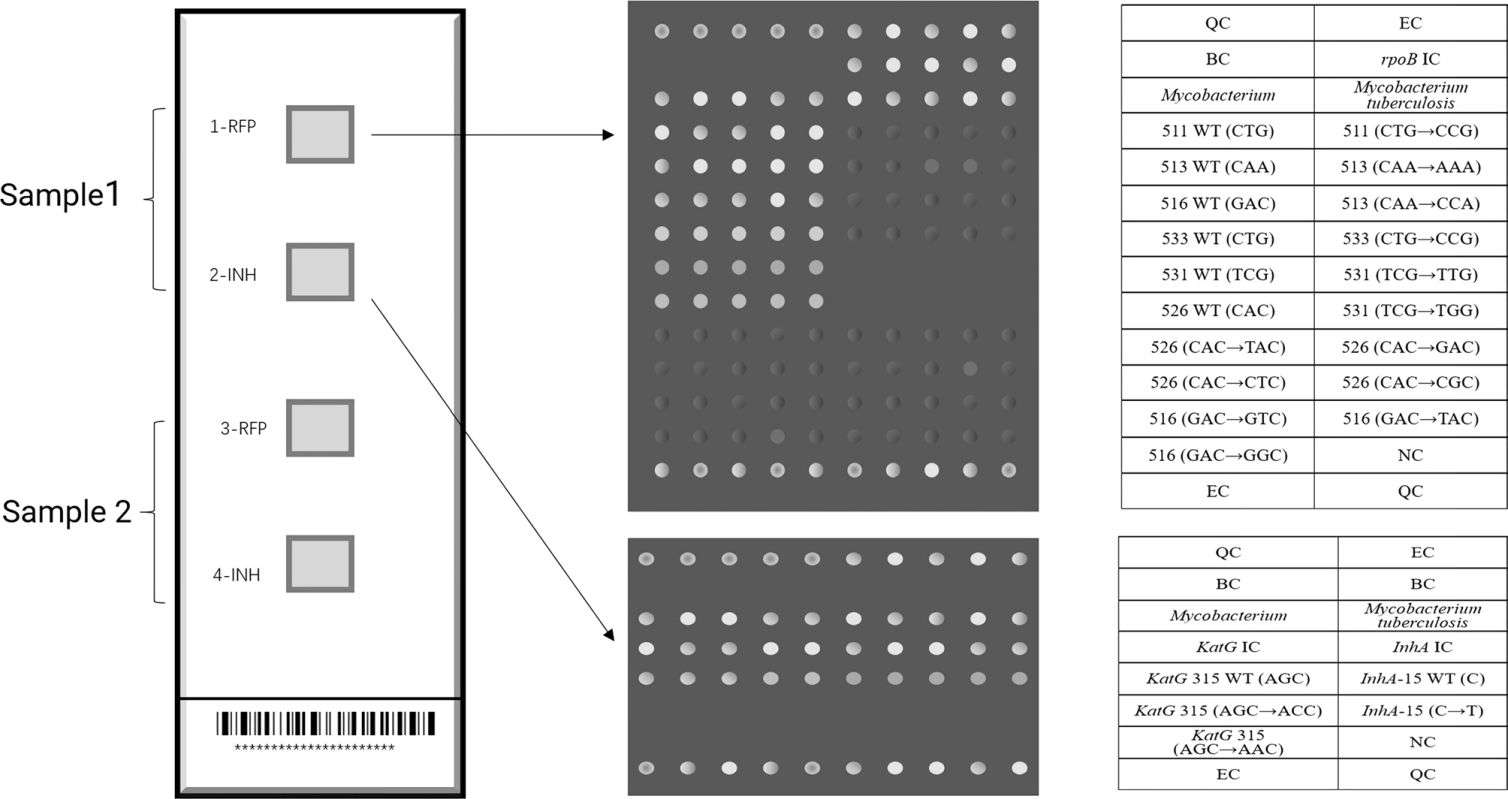
The layout of the DNA microarray method module; each detection panel includes 4 detection modules, which can detect two specimens at the same time. Modules 1 and 3 are used to detect mutations in the *rpoB* gene, and modules 2 and 4 are used to detect mutations in the *katG* gene and *inhA* promoter. *QC* quality control probe; *EC* external control probe; *BC* blank control; *NC* negative control probe; *IC* internal control probe; *WT* wild-type.

The CapitalBio DNA microarray chip method is used to qualitatively detect nucleic acids in samples of *Mtb* isolates from clinical TB patients. It can detect the resistance of samples to RIF and INH within 6 h in full and provide the corresponding gene mutations at the same time. The detection indicators include 3 genes related to resistance to RIF and INH: wild-type and different mutant types of the *rpoB* gene, *katG* gene, and *inhA* gene promoter. Among them, the *rpoB* gene is related to RIF resistance, and a total of 13 mutations are detected at 6 codons, including codon 531 TCG → TGG (Ser531Trp) and TCG → TTG (Ser531Leu); codon 526 CAC → GAC (His526Asp), CAC → TAC (His526Tyr), CAC → CTC (His526Leu), and CAC → CGC (His526Arg); codon 511 CTG → CCG (Leu511Pro); codon 513 CAA → CCA (Gln513Leu) and CAA → AAA (Gln513Lys); codon 516 GAC → GTC (Asp516Val), GAC → TAC (Asp516Tyr), and GAC → GGC (Asp516Gly); and codon 533 CTG → CCG (Leu533Pro). For the INH resistance-related genes, the *katG* gene and *inhA* gene promoter, one gene codon was examined for each: two mutations in codon 315 of the *katG* gene, AGC → ACC (Ser315Thr) and AGC → AAC(Ser315Asn), and the *inhA* gene promoter codon -15 C → T mutant (Fig. 1).

Lianyungang is a city located in eastern China and northern Jiangsu Province. It has a high population density (approximately 703 people/km^2^) and high population mobility. According to a report issued by the local Center for Disease Control and Prevention (CDC), the prevalence of TB in Lianyungang City from 2008 to 2010 was 51.49, 53.26, 55.83 per 100,000 people, and the incidence of drug-resistant TB has also been increasing annually. Therefore, the early diagnosis of drug-resistant TB is very important; thus, with the help of the Jiangsu Provincial Government, the Fourth People’s Hospital of Lianyungang City introduced the CapitalBio DNA microarray chip method in 2010.

In this study, we used long-term and large-scale retrospective analysis to illustrate the effects of the application of DNA microarray technology in the Lianyungang area. In addition, in many previous studies, many others have described the diagnostic efficiency of MTBDRplus, Xpert MTB/RIF, and other methods for different grades of positive smears for acid-fast bacilli^20–22^, but there has been no comprehensive analysis of the gene chip method in these conditions. Therefore, in this study, we evaluated the performance of a gene chip method for sputum smears with different grades of AFB to explore the optimal conditions for this method. Through the analysis of all gene mutation characteristics of drug-resistant TB, the epidemic characteristics of drug-resistant TB bacteria in the Lianyungang area were explored.

## Results

### Gene chip method and the DST

In a sample of 4148 cases, the CapitalBio DNA microarray method showed that 320 were resistant and 3828 were sensitive to RIF, and 342 were resistant and 3716 were sensitive to INH, of which 181 were MDR-TB. Meanwhile, in the DST results, 280 cases were resistant to RIF, and 438 cases were resistant to INH, of which 202 cases were MDR-TB.

### Comparison between the DNA microarray method and DST

The drug resistance phenotype results obtained in traditional drug sensitivity experiments were used as reference standards. The overall sensitivity, specificity, agreement rate (AR), positive predictive value (PPV), negative predictive value (NPV), and k values of the microarray method for RIF resistance detection were 81.4%, 97.6%, 96.5%, 71.3%, 98.6%, and 0.74, respectively. The values for INH resistance detection were 74.0%, 97.1%, 94.7%, 75.0%, 96.9%, and 0.72. The values for MDR-TB are 69.8%, 99.0%, 97.6%, 78.3%, 98.5%, and 0.73. In addition, we compared the diagnostic efficacy of each drug sensitivity test according to the different AFB grades of the sputum smear results (Table 1).

**Table 1:** The drug susceptibility test was used as a standard method to evaluate the efficacy of the DNA microarray for detecting RIF and INH resistance and MDR-TB.

CapitalBio DNA microarray	DST (n = 4148)		Sensitivity (%)	Specificity (%)	AR (%)	PPV (%)	NPV (%)	k
	R	S						
**RIF**								
Overall								
R	228	92	81.4	97.6	96.5	71.3	98.6	0.74
S	52	3776						
≤ AFB 1 +								
R	53	32	72.6	97.7	96.5	62.4	98.6	0.65
S	20	1399						
AFB 2 +								
R	85	36	84.2	95.0	93.7	70.3	97.7	0.73
S	16	690						
AFB 3 +								
R	44	13	84.6	98.2	97.2	77.2	99.2	0.79
S	8	695						
AFB 4 +								
R	46	11	85.2	98.9	98.2	80.7	99.1	0.82
S	8	992						
**INH**								
Overall								
R	324	108	74.0	97.1	94.7	75.0	96.9	0.72
S	114	3602						
≤ AFB 1 +								
R	113	40	67.3	97.0	93.7	73.9	95.9	0.67
S	55	1296						
AFB 2 +								
R	80	25	74.8	96.5	93.7	76.2	96.3	0.72
S	27	695						
AFB 3 +								
R	55	22	76.4	96.8	94.9	71.4	97.5	0.71
S	17	666						
AFB 4 +								
R	76	21	83.5	97.8	96.6	78.4	98.4	0.79
S	15	945						
**MDR-TB**								
Overall								
R	141	39	69.8	99.0	97.6	78.3	98.5	0.73
S	61	3907						
≤ AFB 1 +								
R	36	14	60.0	99.0	97.5	72.0	98.4	0.64
S	24	1430						
AFB 2 +								
R	39	14	70.9	98.2	96.4	73.6	97.9	0.70
S	16	758						
AFB 3 +								
R	31	8	73.8	98.9	97.5	79.5	98.5	0.75
S	11	710						
AFB 4 +								
R	35	3	77.8	99.7	98.8	92.1	99.0	0.84
S	10	1009						

*DST* drug sensitivity test, *R* resistant, *S* susceptible, *AR* agreement rate, *PPV* positive predictive value, *NPV* negative predictive value, *AFB* acid-fast bacilli.

### Information on the mutated codons of various resistance genes

Among all samples resistant to RIF where mutations in the *rpoB* gene were detected, codon 531 mutations accounted for 45.3%, codon 526 mutations accounted for 20.3%, codon 511 mutations accounted for 9.4%, codon 516 mutations accounted for 10.0%, and two or more simultaneous mutations accounted for 8.4% (Table 2).

**Table 2:** The *rpoB* gene mutation of 320 RIF-resistant *Mtb* strains.

Mutant codon(s)	Mutation type	Number	Frequency (%)
511	Leu511Pro	30	9.4
513	Gln513Leu	2	0.6
	Gln513Lys	4	1.3
516	Asp516Val	23	7.2
	Asp516Tyr	6	1.9
	Asp516Gly	3	0.9
526	His526Asp	20	6.3
	His526Tyr	29	9.1
	His526Leu	8	2.5
	His526Arg	8	2.5
531	Ser531Leu	136	42.5
	Ser531Trp	9	2.8
533	Leu533Pro	15	4.7
511, 513	Leu511Pro, Gln513Leu	1	0.3
511, 516	Leu511Pro, Asp516Gly	2	0.6
511, 526	Leu511Pro, His526Tyr	4	1.3
	Leu511Pro, His526Asp	1	0.3
516, 526	Asp516Gly, His526Leu	2	0.6
	Asp516Gly, His526Asp	3	0.9
	Asp516Gly, His526Tyr	1	0.3
516, 531	Asp516Val, Leu533Pro	3	0.9
516, 533	Asp516Gly, Ser531Leu	1	0.3
526, 531	His526Asp, Ser531Leu	2	0.6
511, 516, 526	Leu511Pro, Asp516Tyr, His526Leu	1	0.3
516, 526, 531	Asp516Gly, His526Asp, Ser531Leu	1	0.3
511, 513, 516 ,526	Leu511Pro, Gln513Leu, Asp516Gly, His526Leu	3	0.9
511, 513, 526, 531	Leu511Pro, Gln513Leu, His526Leu, Ser531Trp	2	0.6
Total		320	100

**Figure 2:**
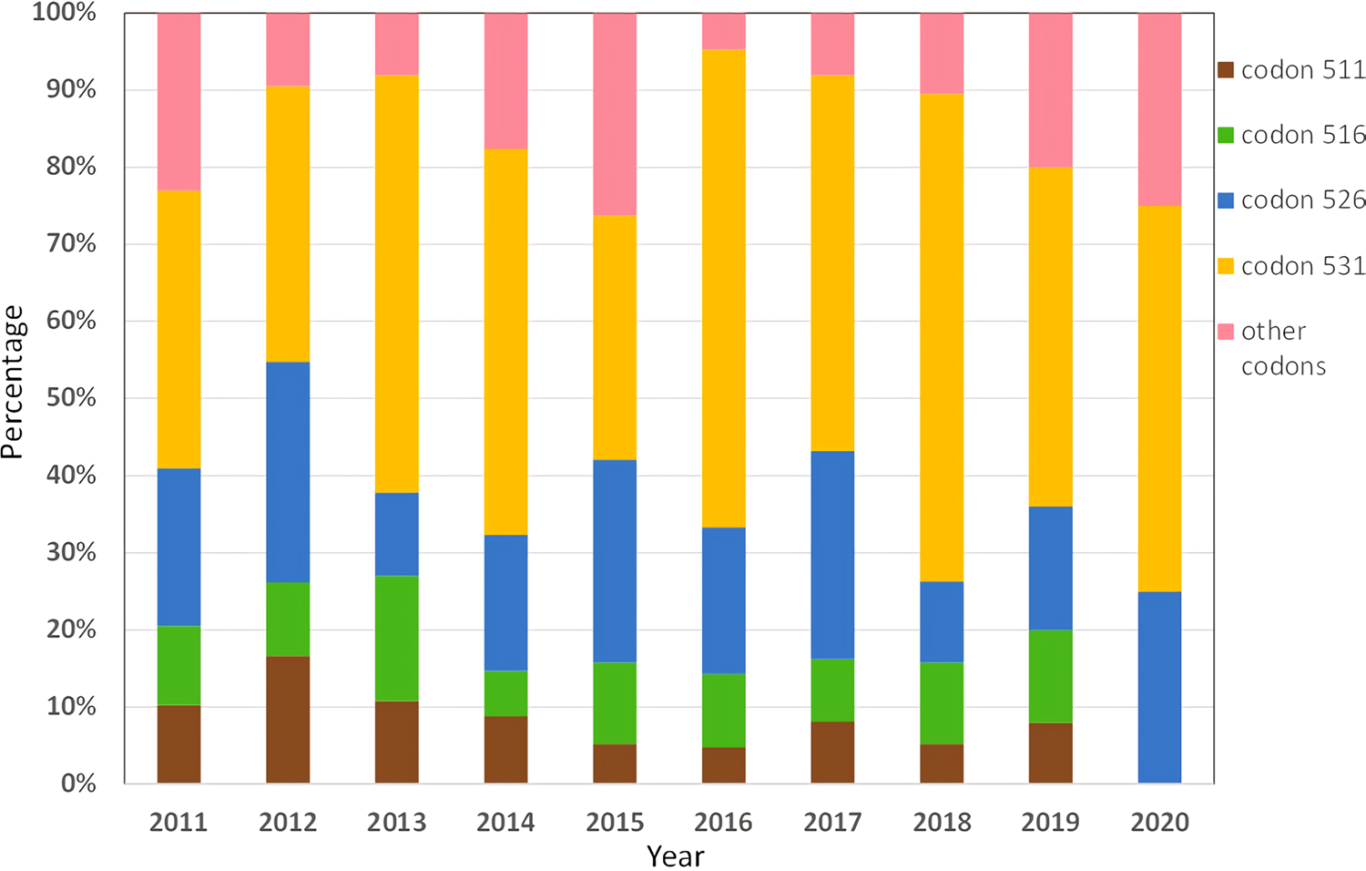
The percentage of major mutation sites in the *rpoB* gene from 2011 to 2020.

We divided the four major mutation codons into four groups, combined all other mutation types into one group, calculated the percentage of various mutation sites each year from 2011 to 2020, and plotted them (Fig. 2), Which is a convenient and intuitive comparison. We found that there was no obvious trend observed for various mutation types over time.

Among all samples resistant to INH, Ser315Thr (*katG*315 AGC → ACC) accounted for 70.5%, Ser315Asn (*katG*315 AGC → AAC) accounted for 4.2%, *inhA*-15 (C → T) accounted for 22.7%, and *katG* plus *inhA* mutations accounted for 2.6% (Table 3).

**Table 3:** The *katG* and *inhA* gene mutations of 320 INH-resistant *Mtb* strains.

Mutant codon(s)	Mutation type	Number	Frequency (%)
*katG*	Ser315Thr	305	70.5
	Ser315Asn	18	4.2
*inhA*	− 15 (C → T)	98	22.7
*katG* + *inhA*	Ser315Thr, − 15 (C → T)	9	0.5
	Ser315Asn, − 15 (C → T)	2	2.1
Total		432	100

## Discussion

INH and RIF are the two most commonly used first-line anti-TB drugs. However, the emergence of drug-resistant strains has severely restricted the use of these two drugs^6^. The most commonly used methods for detecting TB mainly include rapid acid staining of sputum smears and the DST method. However, the sputum smear method can only be used for the preliminary screening test, while the culture and DST method is still the gold standard for diagnosing TB; however, due to the slow growth of this bacteria, isolation may take up to several weeks^23^. To meet the demand for the rapid and accurate detection of *Mtb* and drug resistance, CapitalBio developed a DNA microarray gene detection system. The CapitalBio DNA microarray method can quickly detect *Mtb* drug resistance within 6 h in full. Compared with traditional *Mtb* culture and DST experiments, it has obvious advantages^24^. This study reports the accuracy of the CapitalBio DNA microarray chip method for detecting the resistance of *Mtb* to RIF and INH in Lianyungang City. The sensitivity and specificity obtained in this study are similar to the results obtained in a previous systematic review of the CapitalBio DNA microarray method^25^.

In our study, the DNA microarray method was used to detect large numbers of clinical samples over a long period of time, and the phenotypic drug resistance results of culture and the DST were used as reference standards. Through comparative analysis, we found that the two methods for detecting TB drug resistance have good consistency (k values of 0.4–0.75). In all specimens, the sensitivity, specificity, AR, PPV, NPV, and k values for detecting RIF-resistant *Mtb* were 81.4%, 97.6%, 96.5%, 71.3%, 98.6% and 0.74, respectively; the values for detecting INH-resistant *Mtb* were 74.0%, 97.1%, 94.7%, 75.0%, 96.9% and 0.72; and the values for detecting MDR-TB were 69.8%, 99.0%, 97.6%, 78.3%, 98.5% and 0.73 (Table 1). Compared with a study by Zhang et al.^26^, which also performed direct experiments with clinical specimens, we obtained similar results. However, compared with studies by Zhu et al.^19^ and Caoili et al.^27^, who used purified cultures for gene chip experiments, our sensitivity for detecting RIF resistance and MDR-TB was lower. In this study, we grouped the specimens again according to the different grades from positive smears of acid-fast bacilli and calculated the sensitivity, specificity, AR, PPV, NPV, and ĸ values of the gene chip method in all subgroups. When comparing different sputum smear AFB grades, we found that the DNA microarray method showed different diagnostic performance for different sputum smear AFB grades. The changes in specificity, AR, and NPV for detecting RIF, INH, and MDR-TB were not associated with the changes in the AFB grade, and sensitivity, PPV, and k value all increased as the AFB grade increased. For all parameters, when the grade of the sample was ≤ AFB 1 +, the chip method had the lowest sensitivity, PPV, and k value. The values were 72.6%, 62.4%, and 0.65 for RIF; 67.3%, 73.9%, and 0.67 for INH; and 60.0%, 72.0%, and 0.64 for MDR-TB. When AFB was 4 +, the sensitivity, PPV, and k values were the highest. The values were 85.2%, 80.7%, and 0.82 for RIF; 83.5%, 78.4%, and 0.79 for INH; and 77.8%, 92.1%, and 0.84 for MDR-TB, respectively. However, there was no significant difference in sensitivity or positive predictive value between adjacent groups (P > 0.05). Previous studies have indicated that if the grade of the sample is ≥ AFB 2 +, the MTBDRplus test will perform best^20,28^. Therefore, we grouped the patients again according to a grade of ≤ AFB 1 + and ≥ AFB 2 + (Table 4). The sensitivity of the DNA microarray to detect RIF resistance at ≤ AFB 1 + and ≥ AFB 2 + was 72.6% and 84.5%, respectively (χ^2^ = 5.086, P = 0.024); the sensitivity to detect INH resistance was 67.3% and 78.2%, respectively (χ^2^ = 6.375, P = 0.012); and the sensitivity to detect MDR-TB was 60.0% and 73.9%, respectively (χ^2^ = 3.890, P = 0.049). The P values were both less than 0.05, and the difference was statistically significant. At the same time, there was no significant difference in specificity after testing (P > 0.05). Moreover, when the sputum smear grade was ≥ AFB 2 + , the consistency of the gold standard for RIF resistance and MDR-TB detection showed "very good agreement" (k > 0.75) with the gene chip method, so we believe that when the grade was ≥ AFB 2 + , the DNA microarray method was more sensitive and accurate. This is consistent with the experimental results obtained by the MTBDRplus method. Gauthier et al. proposed a new algorithm for the diagnosis of drug-resistant TB from the perspective of the economic burden. Their research showed that when ≥ AFB 2 +, MTBDRplus is faster and cheaper than liquid-based tests and is the preferred method for the rapid detection of MDR-TB^22^. Our research suggests that this algorithm may also be used for the CapitalBio DNA microarray method, but further research is needed to confirm this hypothesis. However, in this study, 36.26% (1504/4148) of samples were sputum smear-positive grade ≤ AFB 1 +. How to improve the accuracy of the rapid detection of TB drug resistance in this population is a question that cannot be ignored.

**Table 4:** Comparison of the diagnostic efficacy of the DNA microarray method when sputum smear grades were ≤ 1 + and ≥ 2 +

CapitalBio DNA microarray	Sensitivity (%)	Specificity (%)	AR (%)	PPV (%)	NPV (%)	k
**RIF**						
≤ AFB 1 +	72.6	97.7	96.5	62.4	98.6	0.65
≥ AFB 2 +	84.5	97.5	96.5	74.5	98.7	0.77
**INH**						
≤ AFB 1 +	67.3	97.0	93.7	73.9	95.9	0.67
≥ AFB 2 +	78.2	97.1	95.2	75.6	97.5	0.74
**MDR-TB**						
≤ AFB 1 +	60.0	99.0	97.5	72.0	98.4	0.64
≥ AFB 2 +	73.9	99.0	97.7	80.8	98.5	0.76

*DST* drug sensitivity test, *R* resistant, *S* susceptible, *AR* agreement rate, *PPV* positive predictive value, *NPV* negative predictive value, *AFB* acid-fast bacilli.

In the six-month standard treatment plan for TB, RIF has become a key component of anti-TB treatment because of its inhibitory effect on bacterial RNA polymerase (RNAP). It is particularly effective in killing semi-dormant or dormant bacilli^29,30^. However, the emergence and prevalence of drug-resistant RIF strains have posed a dilemma for TB control. The drug resistance of *Mtb* is mainly caused by mutations rather than gene transfer from other bacteria via mobile genetic elements^31^. According to reports, mutations in the *rpoB* gene are the main cause of resistance to RIF in *Mtb*^8,32^. In this study, we found that the most common mutant codons were 531 (45.3%), 526 (20.3%), 516 (10.0%) and 511 (9.4%), and the most common types of mutations included Ser531Leu (42.5%), Leu511Pro (9.4%), His526Tyr (9.1%) and Asp516Va (7.2%) (Table 2). These results are similar to those of previous research reports^31,33–35^. In addition, we detected all 13 *rpoB* mutation types that can be detected with the CapitalBio DNA microarray method; among them, Gln513Leu, His526Arg, and Asp516Gly were rarely reported in previous studies^26,36^, which illustrates the diversity of the gene pool of RIF-resistant TB strains in Lianyungang. In future studies, we should pay close attention to the prevalence of these mutant strains.

On the other hand, INH is one of the common anti-TB drugs used to treat and prevent TB. The leading mechanism of INH resistance is a mutation in *katG*, which encodes an INH activator, and the second most common mechanism of INH resistance is a mutation in the *inhA*-15 (C → T) promoter region, which leads to *inhA* overexpression and titration of the drug^37^. Therefore, the CapitalBio DNA microarray method also detects the mutation of these two genes to determine the resistance to INH. In our study, Ser315Thr (AGC → ACC) was the most common mutation type, accounting for 70.5% of the total detections. The *inhA* − 15 (C → T) promoter mutation accounted for 22.7%, which was the second most common type of mutation. This is similar to the results of others' research^38–40^. It is worth noting that among 432 INH-resistant strains, we found 20 cases of Ser315Asn (AGC → AAC) mutations (including two *katG* 315 + *inhA* mutations), which has rarely been reported in previous studies^26^. This may be attributed to regional differences but also reflects the diversity of drug-resistant bacteria in the region.

There are also limitations to this work. First, because of the difficulty of obtaining samples from patients with extrapulmonary TB, this study focused on sputum specimens only. Second, all the samples included in this study were sputum smear-positive for acid-fast bacilli; we did not collect sputum smear-negative patients with a clinical diagnosis of active TB. According to this study, we found that the CapitalBio DNA microarray chip method has some shortcomings. The sensitivity of the DNA microarray method to detect the resistance to RIF and INH was 81.43% and 73.97%, respectively. This shows that if this method is used alone, there will be a certain degree of risk of missed detection. In addition to the errors that occur during operation, the DNA microarray method itself also has certain shortcomings. Soumitesh Chakravorty et al. used the Xpert MTB/RIF Ultra method to detect 25 types of *rpoB* gene mutations^41^. In addition to the *katG* gene and *inhA* promoter, the mutant genes that cause INH resistance also include at least 23 genes, such as *ahpC, kasA, ndh, iniABC, fadE, furA, Rv1592c* and *Rv1772*^39,42,43^. Therefore, the CapitalBio DNA microarray method also needs to increase the detection range to increase the detection rate of drug-resistant TB. Moreover, compared with traditional culture and drug sensitivity experiments, this method requires sophisticated equipment and highly specialized technical personnel, which also results in only a few areas where this method can be carried out.

Nevertheless, the CapitalBio DNA microarray method is still a very suitable method for detecting the drug resistance of *Mtb*. This study confirmed that this method can directly detect the target gene in clinical specimens with a complex composition. Compared with traditional culture and the DST, this method reduces the testing time required from 8 weeks to 6 h, so it can allow clinical adjustments to the medication plan in time. Since the DST uses live bacteria, it must be carried out in a BLS-3 laboratory. The DNA microarray method significantly reduces the risk of biohazards after the thermal lysis step, allowing it to be performed in a BLS-2 laboratory^22^.

## Conclusion

We used the CapitalBio DNA microarray chip method to detect 4148 clinical specimens with sputum smear positivity for acid-fast bacilli from Lianyungang City. Among them, the Ser531Leu mutation of the *rpoB* gene is the main cause of the resistance of *Mtb* to RIF, and the Ser315Thr mutation of the *katG* gene is the main reason for the resistance of *Mtb* to INH. Through comparisons with the results of the drug sensitivity test (DST), we confirmed that this method is an efficient, accurate, and rapid method for diagnosing the drug resistance of TB, which is very suitable for the direct detection of clinical specimens. The detection efficiency of clinical specimens with a sputum smear grade ≥ AFB 2 + was very good. In summary, this study will help clinicians choose more reasonable testing methods and reduce the economic burden on the government and patients.

## Materials and methods

### Ethics statement

The Ethics Committee of the Fourth People's Hospital of Lianyungang City approved the study (Lianyungang, China [approval number: 2021008]), and informed consent was waived by the ethics committee due to the retrospective nature of the study (Lianyungang, China [approval number: 2021009]). Except for the experimental results, the personal information of all participants was kept confidential. At the same time, we confirmed that all methods were implemented by the industry standards and regulations of China.

### Clinical specimens

**Figure 3:**
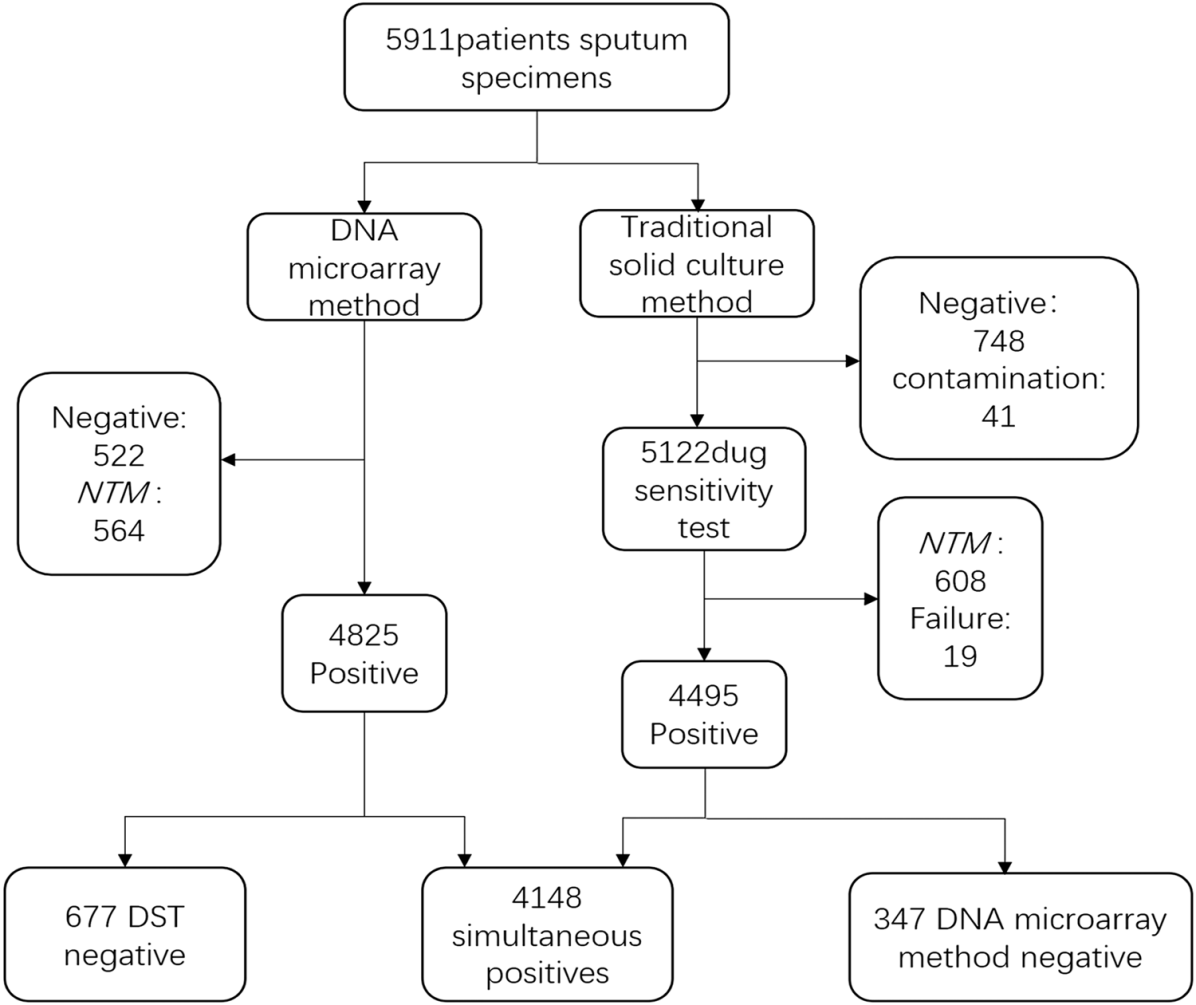
Specimen processing procedure: A total of 5,911 sputum smear-positive specimens were collected. After experimental processing, 4148 specimens that were positive with the DNA microarray method and DST were finally included in the study. *NTM, nontuberculous mycobacteria*; DST, drug sensitivity test.

From January 2010 to December 2020, all sputum specimens with positive sputum smear test results from five hospitals in Lianyungang were sent to the TB laboratory of the Fourth People's Hospital of Lianyungang City. The five hospitals are Donghai County People's Hospital, Guanyun County People's Hospital, Guannan County People's Hospital, Ganyu District People's Hospital, and the Fourth People's Hospital of Lianyungang City. A total of 5911 clinical specimens were obtained, and all sputum specimens were cultured and tested by gene chips at the same time. A total of 5163 specimens had positive culture results. We stained the cultures with acid-fast stains and observed them with a microscope. The observation results of 41 cultures were negative and marked as “contamination”. The remaining 5122 cultures that were positive for acid-fast staining were tested for traditional drug sensitivity. Among them, 608 cultures were identified as *nontuberculous mycobacteria* (*NTM*), and 19 had no drug susceptibility test results. Meanwhile, there were 522 negative and 564 *NTM* in the samples tested by the gene chip method. As a comparison, we included 4148 samples with results from both methods in the study (Fig. 3).

### Traditional culture and drug sensitivity test

Solid culture method: The sputum specimen was treated with an N-acetyl-L cysteine-NaOH (NALC-NaOH) digestion solution, neutralized with phosphate buffer solution, and centrifuged. Then, the phosphate buffer solution was removed by pouring, and the bacterial solution was resuspended in 2 ml. A total of 0.1 ml of sample digestion solution was inoculated in 2 Roche Neutral Solid Medium (BASO, Guangdong, China) and incubated in a 37 °C incubator, and the results were observed weekly. If the strain was growing, the strain was smeared, and a duplicate sample was stained with the acid-fast staining method. If it was positive, the strain was used for the drug sensitivity test; if it was negative, it was considered “contamination”. If the culture result was negative after 8 weeks, it was considered "culture-negative".

Drug susceptibility test: Using a BASO *Mycobacterium* Drug Sensitivity Roche Medium Kit, the culture-positive isolated strains were ground into a 1 mg/ml suspension by the ratio method, and then diluted to 10^–2^ mg/ml and 10^–4^ mg/ml, respectively. A 22 SWG standard inoculation loop was used to pick a full loop (i.e., 0.01 ml) of the suspension and inoculate it into the medium containing RIF and INH. The same method was used to inoculate the control medium without drug and the identification medium containing P-nitrobenzoic acid (PNB). After 4 weeks of continuous incubation at 36 ± 1 °C, the results were observed. If there was no growth on the control medium, it was judged as a "DST failure"; if there was colony growth on both the control medium and PNB medium, then the result was "*NTM*". If there was colony growth on the control group but no colony growth on the PNB medium, the result was "*Mtb*". The number of colonies on the medium was counted, and the resistance rate was calculated as follows: resistance rate (%) = (number of colonies grown on the drug-containing medium/number of colonies grown on the control medium) × 100%. A resistance rate < 1% was considered sensitive, and a resistance rate > 1% was considered resistant.

### CapitalBio DNA microarray chip method

**Figure 4:**
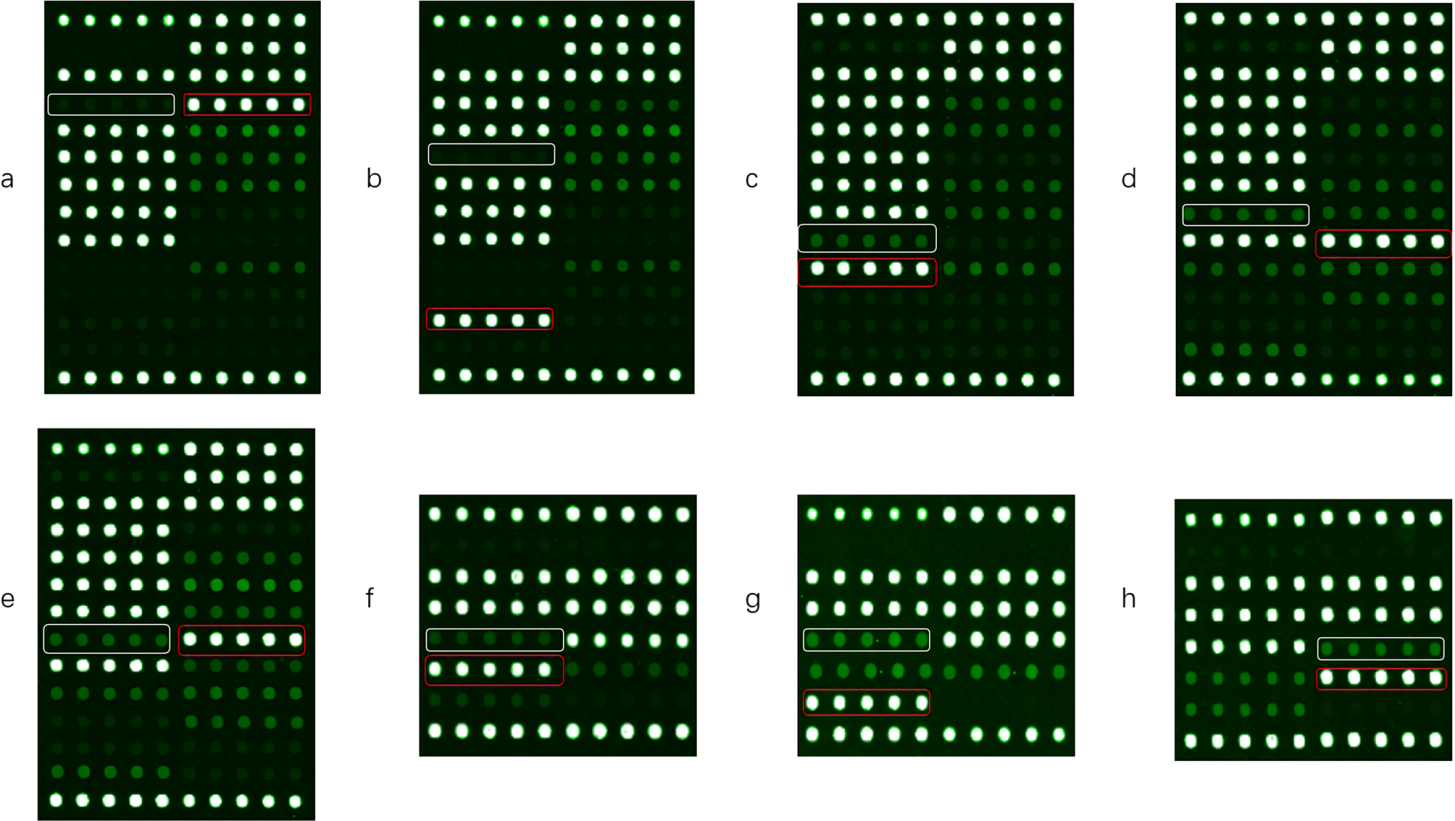
Pattern diagrams of several common drug-resistant gene mutations detected by the DNA microarray method. The white box is the detection site of the wild-type codon, and the red box is the site of the detected mutant codon. (**a**) *rpoB* gene Leu511Pro (CTG → CCG); (**b**) *rpoB* gene Asp516Tyr (GAC → TAC); (**c**) *rpoB* gene His526Tyr (CAC → TAC); (**d**) *rpoB* gene Ser531Trp (TCG → TGG); (**e**) *rpoB* gene Ser531Leu (TCG → TTG); (**f**) *katG* gene Ser315Thr (AGC → ACC); (**g**) *katG* gene Ser315Asn (AGC → AAC); (**h**) *inhA* gene promoter-15 (C → T).

One millilitre of sputum sample was added to 1–2 times 4% sodium hydroxide solution, vortexed, and shaken for 1 min to perform sputum digestion and mixing. A total of 1.0 ml of the digestion solution treated with 4% sodium hydroxide was added to a 1.5 ml centrifuge tube and centrifuged at 12,000 r/min for 5 min. The supernatant was discarded, 1 ml of pH 6.8 phosphate buffer was added, and the sample was shaken and mixed. After homogenization, it was centrifuged at 12,000 r/min for 5 min, and the supernatant was discarded. A total of 80 μl nucleic acid extraction solution was added, and the sample was mixed thoroughly, transferred to a nucleic acid extraction tube, vortexed and shaken to mix well, and placed in an ultrasonic oscillator for 5 min. It was incubated in a dry bath at 95 °C for 15 min, centrifuged at 12,000 r/min for 1 min, and set aside. After following the kit instructions for PCR amplification, chip washing, drying, and chip hybridization, a LuxScan 10K-B microarray chip scanner was used to scan and automatically interpret the results (Fig. 4).

### Statistical analyses

For data analysis, the phenotypic resistance result obtained by the DST was used as a reference standard to calculate the sensitivity, specificity, agreement rate, positive predictive value (PPV), and negative predictive value (NPV) of the CapitalBio DNA microarray. A chi-squared test or two-tailed Fisher’s exact test was used for statistical analysis, and the difference was considered significant when P < 0.05. The degree of agreement between the DST and the GeneChip assay was also assessed using Cohen’s kappa (k) coefficient. k values > 0.75 indicate that the two methods show very good agreement, and k values of 0.40–0.75 show that the two methods show fair to good agreement. k values of < 0.40 indicate that the two methods show poor agreement. All statistical analyses were performed with SPSS 24.0 (IBM Corp, Armonk, NY, USA).

## Supplementary Information

Supplementary Information 1.

Supplementary Information 2.

Supplementary Information 3.

## Data Availability

We have reported all findings in the manuscript. The specimen data, the strains of *Mycobacterium tuberculosis* analyzed in the study, and the original test results of the gene chip can all be obtained from the Tuberculosis Laboratory of the Fourth People's Hospital of Lianyungang City, China. If anyone wants to view or use our mycobacterial strains or our data set, they should contact the corresponding author.
